# Effects of Different Exogenous Insulin Analog Treatments on the Evaluation of Pancreatic Islet Function in Diabetes

**DOI:** 10.1155/jdr/2572581

**Published:** 2026-09-26

**Authors:** Yi-fang Ma, Qiong-li Neng, Yang Ou, Yi-kun Zhou

**Affiliations:** ^1^ Department of Endocrinology and Metabolism, The First People′s Hospital of Yunnan Province, Kunming University of Science and Technology Affiliated Hospital, Kunming, China

**Keywords:** diabetes, electrochemiluminescence immunoassay, exogenous insulin, pancreatic islet function

## Abstract

**Aims:**

Most current domestic and international studies on the effects of different exogenous insulin analogues on insulin detection results have been conducted through in vitro experiments, while relevant in vivo studies are rarely reported. Therefore, this study investigated the impact of different exogenous insulins on insulin detection levels in diabetic patients through in vivo experiments, analyzing the interference of various exogenous insulins with insulin detection by the electrochemiluminescence immunoassay (ECLIA).

**Methods:**

A total of 495 diabetic patients hospitalized in the Department of Endocrinology and Metabolism at the First People′s Hospital of Yunnan Province from January 2018 to October 2025 and undergoing pancreatic islet function testing (using a steamed bun meal test) were selected as study subjects. All subjects met the criterion of fasting C‐peptide (FCP) level < 0.2 nmol/L. Based on the hypoglycemic regimen administered the day before the steamed bun meal test, all subjects were divided into five groups: the continuous subcutaneous insulin infusion (CSII) groups (insulin aspart [aspart] and insulin lispro [lispro]) and the subcutaneous insulin injection at bedtime groups (insulin glargine [glargine], insulin degludec [degludec], and insulin detemir [detemir]). Data was collected on the following characteristics for the five patient groups: gender, age, disease duration, height, weight, body mass index (BMI), and diabetes‐related antibody test results (including IAA, IA‐2A, ICA, GADA, and ZnT8). Measure C‐peptide (CP) and insulin levels in fasting blood samples and in samples taken 0.5, 1, 2, and 3 h after a steamed bun meal using an ECLIA. Also measure fasting blood glucose (FBG) and 2‐h postprandial blood glucose (PBG) on the day of islet function testing. Record the exogenous insulin dose (EINS) and the EINS/weight, as well as the proportion of fasting insulin (INS0h) > 2.5 * μ*IU/mL. A statistical analysis was performed.

**Results:**

There were no significant differences among the five groups in terms of gender, age, height, weight, BMI, disease duration, FBG, PBG, FCP, C‐peptide 0.5 h postprandial (CP0.5h), C‐peptide 1 h postprandial (CP1h), C‐peptide 2 h postprandial (CP2h), C‐peptide 3 h postprandial (CP3h), FCP/FBG, CP2h/PBG, EINS, and EINS/weight (*p* > 0.05). There were no statistically significant differences in diabetes‐related antibody positivity rates among the five groups (*p* > 0.05). INS0h/FBG and INS2h/PBG in the glargine group were significantly higher than in the other four groups (*p* < 0.05). INS0h, insulin levels at all time points after the steamed bun meal, the area under the insulin curve (AUC‐INS), and the proportion of INS0h > 2.5 * μ*IU/mL were all significantly higher in the glargine group than in the other four groups (*p* < 0.05). INS0h in the glargine group showed a positive correlation with EINS/weight levels (*R* = 0.2959, *p* < 0.001).

**Conclusion:**

Insulin glargine can be detected by ECLIA, which causes significant positive interference in insulin test results. The degree of interference positively correlates with EINS/weight. For patients using insulin glargine, INS0h test results may reflect the sum of suppressed endogenous insulin and exogenous insulin. In contrast, ECLIA is virtually incapable of detecting aspart insulin, lispro insulin, deglutamate insulin, and detemir insulin; these analogs exhibit low cross‐reactivity with the ECLIA detection antibodies, resulting in underestimation of insulin levels. Consequently, the true basal level of pancreatic islet function in patients using these insulins may be underestimated.

## 1. Introduction

Diabetes mellitus (DM) is a chronic metabolic disorder resulting from the interaction of multiple factors, including genetics, individual behavior, and environment. It is primarily characterized by hyperglycemia [1, 2]. The clinically common types are Type 1 diabetes mellitus (T1DM) and Type 2 diabetes mellitus (T2DM). Differentiation between T1DM and T2DM can be achieved through results of islet autoantibodies, oral glucose tolerance test (OGTT), insulin release test (IRT), and C‐peptide release test (CPRT) [3–5]. Currently, multiple methods exist for evaluating pancreatic islet function. However, among these approaches, the most direct measure of improved islet cell function remains endogenous insulin secretion itself. In individuals not receiving exogenous insulin therapy, measuring serum or plasma immunoreactive insulin remains the standard method for assessing pancreatic islet cell function [6].

However, due to the hepatic first‐pass effect of endogenous insulin and its physiological characteristics [7, 8], as well as interference from insulin antibodies, using immunoreactive insulin in serum or plasma as an assessment method for pancreatic islet cell function becomes less reliable [9–14]. Therefore, in patients receiving insulin therapy, C‐peptide (CP) evaluation testing should be prioritized. Even in patients not using insulin therapy, peripheral CP levels more accurately reflect portal insulin levels than peripheral insulin measurements [8]. However, CP measurement still has limitations, such as endogenous CP secretion potentially being influenced by exogenous insulin [15–18]. In clinical practice, when assessing pancreatic islet function in Type 1 diabetes patients, some individuals exhibit low CP levels yet detectable high serum insulin concentrations. This suggests that different exogenous insulin analogues may be detected, potentially influencing the evaluation of pancreatic islet function.

Reports on the interference of exogenous insulin analogues with the electrochemiluminescence immunoassay (ECLIA) for insulin detection have been inconsistent [19–21]. Most studies have been conducted solely through in vitro experiments, detecting interference by adding exogenous insulin to serum. However, these insulin analogues may alter cross‐reactivity in assays during in vivo processes such as absorption, distribution, metabolism, and excretion [22]. Additionally, additives potentially present in exogenous insulin preparations may influence in vitro experiments, while their low concentrations in vivo can lead to false elevations or reductions [23]. This study employed in vivo testing by selecting diabetic patients with absolute endogenous insulin deficiency (CP < 0.2 nmol/L) who were treated with different exogenous insulin analogues. This extreme model effectively eliminated interference from endogenous insulin on test results while simulating real clinical conditions by using actual human subjects to reflect true insulin levels. This allowed us to observe the effects of different exogenous insulins on insulin detection levels in diabetic patients, analyze the interference of different exogenous insulins on insulin detection via ECLIA in vivo, identify exogenous insulins that are detectable by this method, and provide guidance for accurately assessing pancreatic islet function in diabetic patients.

## 2. Objects and Methods

### 2.1. Research Object

We selected 495 diabetic patients who were hospitalized in the Department of Endocrinology and Metabolism at the First People′s Hospital of Yunnan Province from January 2018 to October 2025 and underwent pancreatic islet function testing (using a steamed bun meal test) to participate in the study. Inclusion criteria were as follows: (1) meeting the 1999 WHO diagnostic criteria for diabetes; (2) receiving basal insulin at bedtime and rapid‐acting insulin with meals, or using a continuous subcutaneous insulin infusion (CSII) pump; (3) fasting C‐peptide (FCP) < 0.2 nmol/ml; and (4) undergoing the steamed bun meal test. Exclusion criteria were as follows: (1) FCP ≥ 0.2 nmol/L; (2) gestational or lactational diabetes; (3) viral hepatitis, cirrhosis, splenomegaly, or hepatic insufficiency; (4) a prior diagnosis of alcoholic liver disease or a history of heavy alcohol consumption; (5) acute or chronic renal insufficiency; (6) hematologic disorders; (7) acute or chronic infections; (8) acute diabetic complications; and (9) use of noninsulin medications that affect fasting blood glucose (FBG). All subjects were divided into five groups based on the hypoglycemic regimen administered the day before the steamed bun meal test, with detailed grouping criteria as follows: (1) aspart group (*n* = 132): patients using insulin aspart via CSII pump, with basal rate and bolus doses adjusted individually; (2) lispro group (*n* = 103): patients using insulin lispro via CSII pump, with similar titration protocol; (3) glargine group (*n* = 144): patients receiving once‐daily subcutaneous insulin glargine injection at bedtime plus rapid‐acting insulin analogs with meals; (4) degludec group (*n* = 70): patients receiving once‐daily subcutaneous insulin degludec injection at bedtime plus rapid‐acting insulin analogs with meals; and (5) detemir group (*n* = 46): patients receiving once or twice‐daily subcutaneous insulin detemir injection plus rapid‐acting insulin analogs with meals. Diabetes‐related autoantibody status (insulin autoantibody [IAA], protein tyrosine phosphatase antibody [IA‐2A], islet cell antibody [ICA], glutamic acid decarboxylase antibody [GADA], and zinc transporter 8 antibody [ZnT8]) was recorded for each group.

### 2.2. Research Methods

#### 2.2.1. Method for Measuring Indicators

All subjects underwent a steamed bun meal test, which is a commonly used standardized meal test in clinical practice in China for evaluating islet function. This is equivalent to a mixed‐meal tolerance test (MMTT) and provides a standardized stimulus for insulin and CP secretion. The test steps are as follows: On an empty stomach in the morning (no caloric intake for over 8 h), they consumed 100 g of a standard flour‐based steamed bun within 10 min. They were permitted to drink a small amount of water. Venous blood samples were collected at different time points: fasting state (defined as 0 h), and 0.5, 1, 2, and 3 h after the first bite of the bun. Each tube contained 2 mL of blood, which was then centrifuged at 3000 rpm for 15 min to separate the serum. Insulin levels were measured in microinternational units per milliliter and CP levels in nanomoles per liter and glucose levels in millimoles per liter. An electrochemical luminescence, fully automated immunoassay analyzer measured CP and insulin levels at each time point (0, 0.5, 1, 2, and 3 h) for the IRT and CPRT. Calculate the area under the insulin curve (AUC‐INS) as follows: INS0h + 2 × INS0.5h + 3 × INS1h + 4 × INS2h + 2 × INS3h ÷ 4. INS0h, INS0.5h, INS1h, INS2h, and INS3h represent the insulin values at fasting, 0.5 h postprandial, 1 h postprandial, 2 h postprandial, and 3 h postprandial. Blood glucose was measured using the Abbott c16000 biochemical analyzer. Insulin and CP were detected using the Roche Cobas e601 analyzer (using ECLIA). The Yahuilong iFlash3000‐A chemiluminescence immunoassay (Yahuilong Biotechnology Co., Ltd., Shenzhen, China) was used to detect ICAs, IAAs, GADA, IA‐2A, and ZnT8. The assay characteristics and positivity criteria were as follows: IAAs: cutoff value ≥ 10.0 IU/mL; anti‐GADAs: cutoff value ≥ 5.0 IU/mL; ICAs: cutoff value ≥ 1.0 IU/mL; anti‐IA‐2As: cutoff value ≥ 5.0 IU/mL; and ZnT8: cutoff value ≥ 5.0 IU/mL. Values above the respective cutoff were considered positive.

#### 2.2.2. Data Collection

Retrospective data collection included the following for five groups: gender, age, disease duration, height, weight, body mass index (BMI), and diabetes‐related antibody test results (including IAA, IA‐2A, ICA, GADA, and ZnT8). Blood samples were collected for insulin and CP levels at fasting and at 0.5, 1, 2, and 3 h postprandial. Venous FBG and 2‐h postprandial blood glucose (PBG) were collected on the day of pancreatic islet function testing. Recorded data included exogenous insulin dose (EINS), EINS/weight, and the proportion of INS0h > 2.5 * μ*IU/mL. The following comparisons were made: (1) baseline characteristics among the five patient groups; (2) differences in insulin levels across time points, AUC‐INS, and the proportion of INS0h > 2.5 * μ*IU/mL within each group; and (3) correlation between INS0h and EINS/weight.

#### 2.2.3. Statistical Methods

We performed statistical analyses using the SPSS 26.0 software program. We expressed normally distributed quantitative data as mean ± standard deviation (*x* ± *s*). We performed one‐way analysis of variance (ANOVA) to compare multiple groups, followed by LSD post hoc testing. Nonnormally distributed quantitative data were expressed as the median (interquartile range). We performed comparisons among multiple groups using the Kruskal–Wallis *H* rank sum test, followed by Bonferroni post hoc testing. We performed correlation analysis using Spearman′s rho. Categorical data were expressed as *n* (%). Intergroup comparisons were performed using the chi‐square test; Fisher′s exact test was applied when *N* ≤ 5. The area under the curve (AUC) for insulin was calculated using the trapezoidal rule. Effect sizes (*R* for correlation, *F* for ANOVA, *H* for Kruskal–Wallis *H* rank sum test, and *X* ^2^ for chi‐square test) were calculated where appropriate to provide measures of clinical significance. Statistical significance was determined at *α* = 0.05 (two‐tailed), with *p* < 0.05 indicating a statistically significant difference. GraphPad Prism software was used for data visualization.

## 3. Result

### 3.1. Comparing Baseline Characteristics Among the Five Groups

A total of 495 diabetic patients with FCP < 0.2 nmol/L were enrolled in this study. The participants were divided into five groups: aspart group (*n* = 132), lispro group (*n* = 103), glargine group (*n* = 144) (including 50 patients on insulin glargine U300, 41 patients on insulin glargine U100, and 53 patients on recombinant insulin glargine; no statistically significant differences existed between groups), degludec group (*n* = 70), and detemir group (*n* = 46). The male‐to‐female ratio across the five groups was analyzed using the chi‐square test and revealed no statistically significant differences in gender distribution (*χ* ^2^ = 4.685, *p* = 0.323). The five groups showed no statistically significant differences in age, height, weight, BMI, disease duration, FBG, PBG, FCP, C‐peptide 0.5 h postprandial (CP0.5h), C‐peptide 1 h postprandial (CP1h), C‐peptide 2 h postprandial (CP2h), C‐peptide 3 h postprandial (CP3h), FCP/FBG, CP2h/PBG, EINS, and EINS/weight (*p* > 0.05). However, INS0h/FBG and INS2h/PBG levels were significantly higher in the glargine group than in the other four groups (*p* < 0.05). No statistically significant differences were observed between the remaining four groups. Detailed results are presented in Table 1.

**Table 1:** Comparison of general characteristics among five groups.

Variable	Aspart (*n* = 132)	Lispro (*n* = 103)	Glargine (*n* = 144)	Degludec (*n* = 70)	Detemir (*n* = 46)	*H*/*F*/*X* ^2^	*p*
Age (years)	38.00 (30.00, 50.00)	44.00 (31.00, 53.00)	44.50 (29.25, 54.75)	45.00 (36.75, 58.00)	37.50 (28.75, 51.50)	5.846	0.211
Height (cm)	164.00 (160.00, 170.00)	165.00 (159.00, 170.00)	163.00 (158.00, 170.00)	167.50 (160.00, 170.75)	163.00 (156.50, 169.00)	4.271	0.371
Weight (kg)	55.00 (49.75, 60.00)	55.00 (50.00, 60.00)	55.00 (48.00, 61.75)	59.50 (50.00, 66.00)	53.00 (48.50, 60.00)	6.276	0.179
BMI (kg/m^2^)	20.32 (18.47, 22.57)	20.37 (18.69, 22.03)	20.53 (18.78, 22.42)	20.94 (18.78, 22.22)	19.95 (18.31, 22.04)	2.532	0.639
Disease duration (years)	4.00 (1.00, 10.00)	3.00 (0.50, 9.00)	5.00 (2.00, 10.00)	4.00 (2.00, 10.00)	5.00 (1.00, 10.00)	8.739	0.068
Gender (male/female)	67/65	60/43	71/73	43/27	22/24	4.685	0.323
FCP (nmol/L)	0.030 (0.001, 0.106)	0.043 (0.006, 0.092)	0.021 (0.001, 0.079)	0.026 (0.004, 0.082)	0.031 (0.003, 0.065)	3.456	0.485
CP0.5h (nmol/L)	0.043 (0.001, 0.142)	0.065 (0.012, 0.117)	0.034 (0.001, 0.109)	0.038 (0.009, 0.109)	0.045 (0.003, 0.104)	4.322	0.364
CP1h (nmol/L)	0.057 (0.001, 0.183)	0.091 (0.012, 0.164)	0.037 (0.001, 0.134)	0.043 (0.001, 0.164)	0.069 (0.005, 0.173)	4.270	0.371
CP2h (nmol/L)	0.071 (0.003, 0.247)	0.108 (0.014, 0.265)	0.043 (0.003, 0.193)	0.078 (0.012, 0.217)	0.093 (0.008, 0.287)	4.932	0.371
CP3h (nmol/L)	0.088 (0.003, 0.291)	0.119 (0.018, 0.314)	0.062 (0.002, 0.225)	0.083 (0.013, 0.299)	0.143 (0.014, 0.306)	6.173	0.187
FBG (mmol/L)	8.10 (6.00, 10.90)	7.05 (5.70, 10.40)	7.40 (5.50, 10.50)	6.45 (4.73, 10.40)	7.35 (5.48, 8.43)	6.901	0.141
PBG (mmol/L)	20.53 ± 5.75	19.53 ± 6.48	19.62 ± 6.24	18.74 ± 5.16	19.34 ± 5.63	1.082	0.365
FCP/FBG	0.0036 (0.0002, 0.0140)	0.0056 (0.0007, 0.0136)	0.0024 (0.0002, 0.0095)	0.0038 (0.0004, 0.0127)	0.0036 (0.0005, 0.0084)	3.833	0.429
CP2h/PBG	0.0037 (0.0001, 0.0135)	0.0665 (0.0113, 0.1578)	0.0028 (0.0002, 0.0096)	0.0056 (0.0008, 0.0133)	0.0063 (0.0003, 0.0147)	4.045	0.400
EINS (IU)	13.85 (10.72, 16.80)	12.00 (9.60, 14.60)	14.00 (10.00, 18.00)	14.00 (10.00, 16.00)	12.00 (10.00, 14.25)	4.440	0.350
EINS/weight (IU/kg)	0.245 (0.202, 0.305)	0.228 (0.194, 0.272)	0.255 (0.207, 0.295)	0.221 (0.186, 0.270)	0.235 (0.213, 0.269)	8.495	0.075
INS0h/FBG	0.041 (0.140, 0.242)^a^	0.026 (0.015, 0.111)^a^	0.424 (0.259, 0.810)	0.035 (0.019, 0.076)^a^	0.055 (0.022, 0.245)^a^	144.689	< 0.001
INS2h/PBG	0.075 (0.008, 0.158)^a^	0.066 (0.011, 0.158)^a^	0.194 (0.105, 0.336)	0.071 (0.009, 0.147)^a^	0.119 (0.019, 0.266)^b^	63.799	< 0.001

*Note:* Data are shown as counts, mean ± standard deviation, and median (lower quartile *P*25 and upper quartile *P*75). All *p* values were two‐tailed distributions, and *p* < 0.05 was considered statistically significant.

Abbreviations: BMI = body mass index, CP0.5h = C‐peptide 0.5 h postprandial, CP1h = C‐peptide 1 h postprandial, CP2h = C‐peptide 2 h postprandial, CP3h = C‐peptide 3 h postprandial, EINS = exogenous insulin dose, FBG = fasting blood glucose, FCP = fasting C‐peptide, INS0.5h = insulin 0.5 h postprandial, INS0h = fasting insulin, INS1h = insulin 1 h postprandial, INS2h = insulin 2 h postprandial, INS3h = insulin 3 h postprandial, PBG = 2‐h postprandial blood glucose.

^a^ *p* < 0.001, compared with the glargine group.

^b^ *p* < 0.05, compared with the glargine group.

### 3.2. Comparison of Insulin Levels Across Different Time Periods for Each Group

After excluding the effects of EINS and EINS/weight levels and finding no statistically significant differences in FBG and PBG levels among the five groups (*p* > 0.05), we observed significant differences in INS0h/FBG and INS2h/PBG levels among the five groups (*p* < 0.05). We employed the Kruskal–Wallis *H* rank sum test to compare insulin levels across the five groups during fasting and at various time points after eating steamed buns. Post hoc multiple comparisons were performed using the Bonferroni test. The results showed that the glargine group had significantly higher levels of INS0h, INS0.5h, INS1h, INS2h, and INS3h than the other four groups, with statistically significant differences (*p* < 0.05). However, no statistically significant differences were observed among the remaining four groups (*p* > 0.05). Detailed results are presented in Table 2.

**Table 2:** Comparison of insulin levels across different time periods for each group.

Variable	Aspart (*n* = 132)	Lispro (*n* = 103)	Glargine (*n* = 144)	Degludec (*n* = 70)	Detemir (*n* = 46)	*H*	*p*
INS0h (*μ*IU/mL)	0.325 (0.100, 1.965)^a^	0.200 (0.100, 0.824)^a^	3.080 (2.128, 4.743)	0.200 (0.100, 0.544)^a^	0.434 (0.100, 2.130)^a^	182.679	< 0.001
INS0.5h (*μ*IU/mL)	0.743 (0.100, 2.448)^a^	0.461 (0.100, 1.445)^a^	3.110 (2.185, 5.228)	0.200 (0.100, 0.877)^a^	1.150 (0.200, 2.730)^a^	130.332	< 0.001
INS1h (*μ*IU/mL)	1.155 (0.100, 3.010)^a^	0.842 (0.200, 2.595)^a^	3.765 (2.248, 5.248)	0.285 (0.200, 1.170)^a^	2.150 (0.200, 3.470)^a^	93.140	< 0.001
INS2h (*μ*IU/mL)	1.420 (0.100, 3.420)^a^	1.145 (0.200, 2.965)^a^	3.605 (2.198, 6.278)	1.095 (0.200, 2.895)^a^	2.390 (0.200, 3.790)^b^	74.457	< 0.001
INS3h (*μ*IU/mL)	1.905 (0.100, 3.343)^a^	1.075 (0.200, 2.600)^a^	3.915 (2.363, 6.480)	1.340 (0.200, 2.895)^a^	2.885 (0.224, 5.285)^c^	73.306	< 0.001

*Note:* Data are shown as median (lower quartile *P*25 and upper quartile *P*75). All *p* values were two‐tailed distributions, and *p* < 0.05 was considered statistically significant.

Abbreviations: INS0.5h = insulin 0.5 h postprandial, INS0h = fasting insulin, INS1h = insulin 1 h postprandial, INS2h = insulin 2 h postprandial, INS3h = insulin 3 h postprandial.

^a^ *p* < 0.001, compared with the glargine group.

^b^ *p* < 0.01, compared with the glargine group.

^c^ *p* < 0.05. compared with the glargine group.

### 3.3. Comparison of the AUC‐INS Among Five Groups

**Figure 1:**
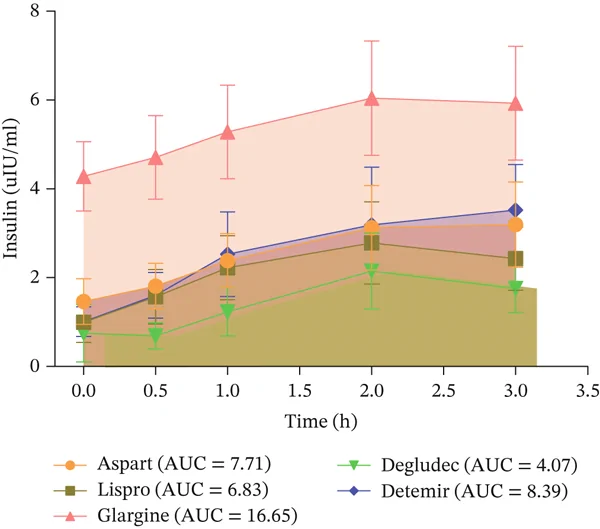
Comparison of AUC‐INS levels across five groups. Note: AUC = area under the curve, AUC‐INS = area under the insulin curve. The insulin levels measured at each time point and the AUC‐INS values in the glargine group were significantly higher than those in the other four groups (*p* < 0.05). All *p* values were two‐tailed distributions, and *p* < 0.05 was considered statistically significant.

We employed the Kruskal–Wallis *H* rank sum test to compare the AUC‐INS across the five groups. The results revealed statistically significant differences in AUC‐INS levels among the five groups (*p* < 0.001). Post hoc multiple comparisons were performed using the Bonferroni test. The results showed that patients in the glargine group had significantly higher AUC‐INS levels than patients in the other four groups (*p* < 0.01). There were no statistically significant differences among the remaining four groups (*p* > 0.05). The results are presented in Figure 1.

### 3.4. Comparison of the Proportion of INS0h > 2.5 * μ*IU/mL Across Five Groups

**Figure 2:**
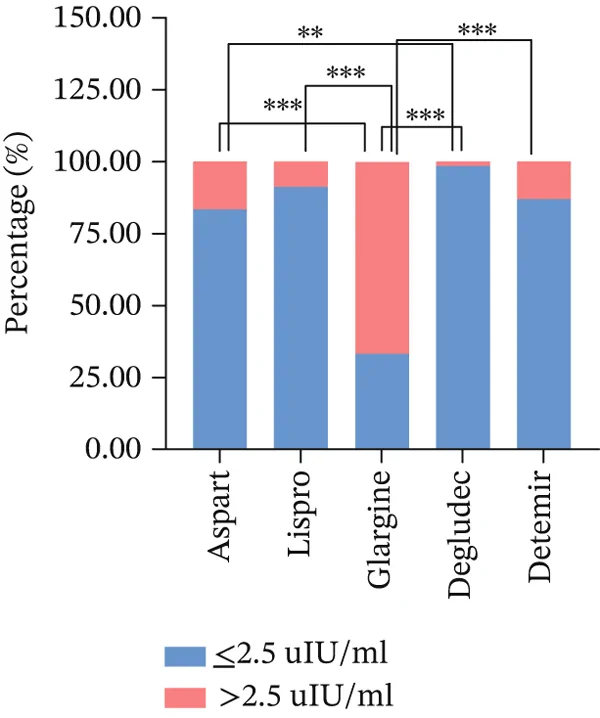
Comparison of the proportion of INS0h > 2.5 * μ*IU/mL across five groups. Note: INS0h = fasting insulin. With an adjusted *α* ^′^ = 0.005 as the significance level, *p* < 0.005 was considered statistically significant.  ^∗∗∗^ *p* < 0.001;  ^∗∗^ *p* < 0.01.

According to relevant studies, when pancreatic islet function is severely impaired (CP < 0.2 nmol/L), the corresponding insulin detection value is < 2.5 *μ*IU/mL [24]. This study used an insulin detection threshold of 2.5 *μ*IU/mL as the critical point. We defined insulin levels ≤ 2.5 * μ*IU/mL as undetectable exogenous insulin and insulin levels > 2.5 * μ*IU/mL as considered detectable exogenous insulin. We performed a chi‐square test on the proportion of INS0h > 2.5 * μ*IU/mL across the five groups. Post hoc pairwise comparisons of multiple sample rates were conducted using the chi‐square partitioning method, with an adjusted *α* ^′^ = 0.005 as the significance level. The results showed that the proportion of INS0h > 2.5 * μ*IU/mL in the glargine group was significantly higher than in the other four groups (*p* < 0.001). Detailed results are presented in Figure 2.

### 3.5. Correlation of INS0h and EINS/Weight Across Five Groups

We employed Spearman′s correlation analysis to examine the correlation between INS0h and EINS/weight in five insulin groups. The results showed that INS0h was not correlated with EINS/weight in the aspart, lispro, degludec, and detemir groups (*p* > 0.05). However, the INS0h was positively correlated with the EINS/weight in the glargine group (*R* = 0.2959, *p* < 0.001). The results are presented in Table 3.

**Table 3:** Correlation of INS0h (microinternational units per milliliter) and EINS/weight (international units per kilogram) in five groups.

Variable	Median (P25, P75)	*R*	*p*
INS0h (aspart)	0.325 (0.100, 1.965)	0.1175	0.1937
EINS/weight (aspart)	0.245 (0.202, 0.305)		
INS0h (lispro)	0.200 (0.100, 0.824)	0.0396	0.6929
EINS/weight (lispro)	0.228 (0.194, 0.272)		
INS0h (glargine)	3.080 (2.128, 4.743)	0.2959	0.0003
EINS/weight (glargine)	0.255 (0.207, 0.295)		
INS0h (degludec)	0.200 (0.100, 0.544)	0.0997	0.4186
EINS/weight (degludec)	0.221 (0.186, 0.270)		
INS0h (detemir)	0.434 (0.100, 2.130)	0.1142	0.4553
EINS/weight (detemir)	0.235 (0.213, 0.269)		

*Note:* Data are shown as median (*P*25 and *P*75). All *p* values were two‐tailed distributions, and *p* < 0.05 was considered statistically significant.

Abbreviations: EINS = exogenous insulin dose, INS0h = fasting insulin.

### 3.6. Comparison of Diabetes‐Related Antibody Positivity Rates Among the Five Groups

We employed the chi‐square test to compare the diabetes‐related antibody (including IAA, IA‐2A, ICA, GADA, and ZnT8) positivity rates across the five groups. The results indicated no statistically significant differences in diabetes‐related antibody positivity rates among the five groups (*p* > 0.05), as shown in Table 4.

**Table 4:** Comparison of diabetes‐related antibody positivity rates among five groups *n* (%).

Group	IAA		IA‐2A		ICA		GADA		ZnT8	
	+	−	+	−	+	−	+	−	+	−
Aspart	14 (11.1%)	112 (88.9%)	4 (7.3%)	51 (92.7%)	13 (10.3%)	113 (89.7%)	31 (24.4%)	96 (75.6%)	0 (0.0%)	16 (100%)
Lispro	15 (14.9%)	86 (85.1%)	5 (8.8%)	52 (91.2%)	11 (11.1%)	88 (88.9%)	29 (29.3%)	70 (70.7%)	1 (4.5%)	21 (95.5%)
Glargine	21 (16.0%)	110 (84.0%)	3 (5.3%)	54 (94.7%)	8 (6.1%)	123 (93.9%)	26 (19.7%)	106 (80.3%)	0 (0.0%)	25 (100.0%)
Degludec	13 (20.6%)	50 (79.4%)	1 (3.0%)	32 (97.0%)	12 (19.0%)	51 (81.0%)	18 (28.6%)	45 (71.4%)	0 (0.0%)	7 (100.0%)
Detemir	3 (8.6%)	32 (91.4%)	2 (6.9%)	27 (93.1%)	4 (11.8%)	30 (88.2%)	6 (17.6%)	28 (82.4%)	0 (0.0%)	10 (100.0%)
*X* ^2^	4.336		1.337		7.464		4.317		3.860	
*p*	0.362		0.878		0.106		0.365		0.688	

*Note:* Data are shown as counts (%). All *p* values were two‐tailed distributions, and *p* < 0.05 was considered statistically significant.

## 4. Discussion

In this study, we investigated the effects of five different exogenous insulin analogs on insulin and CP measurements using ECLIA in 495 diabetic patients with severe *β*‐cell dysfunction (FCP < 0.2 nmol/L). Our main findings indicate that insulin glargine shows significant cross‐reactivity with the ECLIA assay, resulting in falsely elevated insulin measurements, while insulin aspart, lispro, degludec, and detemir exhibit minimal to no detectable cross‐reactivity. The degree of interference from insulin glargine positively correlates with the EINS/weight.

Studies indicate that during hypoglycemia in exogenous insulin autoimmune syndrome (EIAS), the concentration of immunologically active insulin in the blood increases significantly, far exceeding the increase in CP. This results in insulin–CP dissociation [15–17]. Research indicates that EIAS patients with hyperinsulinemia exhibit low CP concentrations, demonstrating a dissociation between the two [18]. This occurs because excessive exogenous insulin release suppresses endogenous insulin secretion, resulting in low CP levels. Additionally, IAA binding to insulin prolongs insulin half‐life and increases its “reserve capacity,” while CP half‐life remains unaffected [25, 26]. Exogenous insulin affects CP similarly to antibody‐bound insulin release, partially inhibiting *β*‐cells insulin secretion and thereby reducing endogenous CP. Consequently, when evaluating CP in diabetic patients with residual pancreatic islet function (e.g., Type 2 diabetes) during exogenous insulin therapy, true *β*‐cell function may be underestimated [27, 28].

Currently, serum insulin detection primarily uses immunoassays that detect insulin by binding insulin‐specific antibodies to insulin. However, insulin antibodies exhibit cross‐reactivity with synthetic human insulin and insulin analogues. Relevant reports indicate that different exogenous insulin analogues, detection methods, and reagents from various manufacturers exert varying effects on insulin detection [29]. ECLIA is one of the most commonly used detection methods today. Manufacturer instructions cite earlier literature that concluded, through in vitro experiments, that three insulin analogues (insulin aspart, insulin lispro, and insulin glargine) do not interfere with insulin detection by ECLIA [29]. However, in clinical practice, some patients using insulin analogues still exhibit discrepancies between CP and insulin levels, even after excluding interference from insulin antibodies. Therefore, we conducted this study to further clarify the impact of exogenous insulin analogues on insulin detection by ECLIA through in vivo experiments.

Human insulin has two antigenic determinants: one between Amino Acids 27 and 30 on the B chain and the other between Amino Acids 8 and 10 on the A chain [30, 31]. This study included five insulin analogues with distinct structural modifications. Insulin aspart replaces the proline at Position 28 of the human insulin B chain with aspartic acid, while insulin lispro swaps the 28th and 29th amino acids on the insulin B chain. The structural modifications in insulin analogs can alter antibody binding affinity by changing the conformation of antigenic epitopes. Analogs with modifications outside the primary antibody binding regions (e.g., B27–30 and A8–10) may retain cross‐reactivity, while those with modifications within or near these epitopes may show reduced detection by immunoassays. Zhang et al. and Owen and Roberts [23, 29]demonstrated through in vitro experiments that insulin lispro and insulin aspart do not interfere with the Roche E170 system (using ECLIA) and that the probability of detecting insulin lispro and aspart is extremely low. Dayaldasani et al. demonstrated in vitro that insulin aspart and insulin lispro cause positive interference in the Advia Centaur system (using direct chemiluminescence) and the Immulite 2000 system (using enzyme‐linked chemiluminescence) [32]. Moriyama et al. demonstrated through in vitro experiments that insulin aspart caused weak interference and insulin lispro exhibited positive interference on the ARCHITECT i2000 system (using direct chemiluminescence) [33]. Additionally, Yoshida et al. [20] demonstrated through in vivo testing that insulin lispro was undetectable in ECLusys assays (using ECLIA), which is consistent with the findings of our study. These findings suggest that differences between in vivo and in vitro environments, as well as the choice of insulin detection method, can influence final measurement results.

Insulin glargine is formed by replacing the aspartic acid at Position 21 on the A chain with glycine and appending two arginine molecules (30a and 30b) to threonine at Position 30 on the C‐terminal of the B chain. After subcutaneous injection, insulin glargine is rapidly degraded upon acid neutralization (pH = 4) into M1 (with aspartic acid at Position 21 on the A chain replaced by glycine and the B chain intact) and M2 (where the threonine at Position 30 on the C‐terminal of the B chain is modified with two arginine molecules, 30a and 30b). M1 exhibits cross‐reactivity with insulin assays, whereas M2 does not [19]. This study demonstrates that insulin glargine causes significant positive interference in insulin detection by ECLIA, consistent with findings from Zhang et al.′s in vitro studies [23]. Similarly, Dayaldasani et al. demonstrated through in vitro experiments that insulin glargine exhibited cross‐reactivity in Roche Elecsys E170 insulin analog assays (using ECLIA), with interference increasing at higher analogue concentrations [32]. However, Owen and Roberts [29] demonstrated in vitro that insulin glargine did not interfere with insulin detection when fetal bovine serum was used as the base serum, indicating that different base sera may produce different results.

Insulin degludec has a 16‐carbon fatty acid side chain that is linked to Lysine 29 of the B chain via an L‐*γ*‐glutamic acid linker, and the threonine residue at Position 30 has been removed. This design enables a unique hexameric reservoir mechanism that forms the molecular basis for its ultralong duration and smooth action profile [34, 35]. Current reports on insulin degludec interfering with insulin detection are scarce. This study demonstrates that insulin degludec is virtually undetectable by ECLIA. Plasma insulin levels only reflect suppressed endogenous insulin.

Insulin detemir has a covalently bound 14‐carbon free fatty acid (myristic acid) side chain at the S‐position of Lysine 29 on the B chain. The threonine residue at Position 30 has been removed. Parfitt et al. [36] conducted in vitro experiments using fasting blood from healthy individuals as the baseline serum. At interference concentrations of 43.2 and 144 *μ*U/mL, insulin detemir showed no interference with ECLIA insulin detection, which is consistent with the findings of this study. Heurtault et al. [19] conducted in vitro experiments using fetal bovine serum (with an insulin concentration of nearly 0 *μ*U/mL) as the baseline serum. At an interference concentration of 200 mU/L, insulin detemir exhibited 0.1% cross‐reactivity. Zhang et al. [23] conducted in vitro experiments using human serum with high and low concentrations as the baseline serum. The results showed low cross‐reactivity of insulin detemir with the high‐concentration base serum and no interference with the low‐concentration base serum.

Since CP and insulin are secreted into the bloodstream in equimolar amounts, CP levels are commonly used as a marker for insulin levels in clinical pancreatic islet function assessments. However, the use of exogenous insulin suppresses endogenous insulin secretion and, consequently, endogenous CP levels. This suppression may lead to an underestimation of CP levels and cause bias in the assessment of pancreatic islet function. Consequently, CP alone may have limitations in certain clinical contexts. Since insulin glargine can be detected via ECLIA, plasma insulin levels may represent the sum of endogenous insulin and insulin glargine. Conversely, insulin aspart, insulin lispro, insulin degludec, and insulin detemir are barely detectable by ECLIA. Consequently, plasma insulin levels reflect endogenous insulin (after exogenous insulin suppression). Because of this suppression, insulin test values are lower than actual levels. Thus, in clinical practice, relying solely on CP levels to assess pancreatic islet function in DM patients treated with exogenous insulin may lead to an underestimation of pancreatic islet function. Additionally, when evaluating fasting insulin levels, it is important to consider the type of exogenous insulin used and the detection method. For example, when using ECLIA to assess patients using insulin glargine, plasma insulin levels may reflect the sum of endogenous insulin and insulin glargine. However, when using insulin aspart, insulin lispro, insulin degludec, or insulin detemir, the detected result represents only suppressed endogenous insulin, which can lead to an underestimation.

This study has certain limitations. First, the sample size was relatively limited, particularly for certain insulin analogues, due to the limited use of insulin detemir (with a shorter half‐life) and insulin degludec (not yet approved for T1DM in China) in patients with complete endogenous insulin deficiency. This may impact the statistical power of the results. Future validation requires multicenter, large‐sample prospective studies. Second, the study only examined the effects of five common exogenous insulin analogues on insulin detection by ECLIA. It did not address interference from other insulin formulations or novel detection methods, such as liquid chromatography–tandem mass spectrometry. Additionally, potential confounding factors include variability in insulin dosing regimens among patients, heterogeneity in diabetes duration and severity, and the potential impact of renal function on CP clearance. Future studies with larger sample sizes and more diverse patient populations are needed to confirm these findings.

## 5. Conclusion

Insulin glargine can be detected by ECLIA, which causes significant positive interference in insulin detection results. The degree of interference is positively correlated with the EINS/weight (*R* = 0.2959, *p* < 0.001). For patients using insulin glargine, fasting insulin detection results may reflect the sum of suppressed endogenous insulin and exogenous insulin. In contrast, ECLIA is virtually incapable of detecting aspart insulin, lispro insulin, deglutamate insulin, and detemir insulin; these analogs exhibit low cross‐reactivity with the ECLIA detection antibodies, resulting in underestimation of insulin levels. Consequently, the true basal level of pancreatic islet function of patients using these types of insulin may be underestimated. Therefore, for diabetic patients receiving exogenous insulin therapy, relying solely on CP levels to assess pancreatic islet function has limitations. A comprehensive evaluation requires considering both the type of exogenous insulin used and the detection method employed. These findings should be interpreted within the context of specific insulin therapies and assay characteristics, and further studies with larger sample sizes are needed to confirm these findings.

**Nomenclature**
ECLIA: electrochemiluminescence immunoassay
FCP: fasting C‐peptide
CSII: continuous subcutaneous insulin infusion
BMI: body mass index
IAA: insulin autoantibody
IA2A: antiprotein tyrosine phosphatase antibody
ICA: islet cell antibody
GADA: antiglutamicacid decarboxylase antibody
ZnT8: zinc transporter 8
CP: C‐peptide
FBG: fasting blood glucose
PBG: 2‐h postprandial blood glucose
EINS: exogenous insulin dose
CP0.5 h: C‐peptide 0.5 h postprandial
CP1h: C‐peptide 1 h postprandial
CP2h: C‐peptide 2 h postprandial
CP3h: C‐peptide 3 h postprandial
AUC‐INS: area under the insulin curve
DM: diabetes mellitus
T1DM: Type 1 diabetes mellitus
T2DM: Type 2 diabetes mellitus
OGTT: oral glucose tolerance test
IRT: insulin release test
CPRT: C‐peptide release test
INS0h: fasting insulin
INS0.5h: insulin 0.5 h postprandial
INS1h: insulin 1 h postprandial
INS2h: insulin 2 h postprandial
INS3h: insulin 3 h postprandial
AUC: area under the curve
EIAS: exogenous insulin autoimmune syndrome

## Author Contributions

Conceptualization: Yi‐kun Zhou and Yang Ou. Methodology: Yi‐kun Zhou, Yi‐fang Ma, and Qiong‐li Neng. Software: Yi‐fang Ma. Validation: Yi‐kun Zhou and Yang Ou. Formal analysis: Yi‐fang Ma and Qiong‐li Neng. Investigation: Yi‐fang Ma and Qiong‐li Neng. Resources: Yi‐kun Zhou and Yang Ou. Data curation: Yi‐fang Ma. Writing—original draft preparation: Yi‐fang Ma, Qiong‐li Neng, and Yi‐kun Zhou. Writing—review and editing: Yi‐kun Zhou and Yang Ou. Visualization: Yi‐fang Ma. Supervision: Yi‐kun Zhou and Yang Ou. Project administration: Yi‐kun Zhou. Funding acquisition: Yi‐kun Zhou. Yi‐fang Ma and Qiong‐li Neng contributed equally to this work and share first authorship.

## Funding

The study was funded by the National Key Clinical Specialty Training Program for Endocrinology–Open Research Topics (2024NMKFKT‐08) and Yunnan Health Training Project of High‐Level Talents (L‐2024006).

## Disclosure

All authors have read and approved the final version of the manuscript. Yi‐kun Zhou had full access to all of the data in this study and takes complete responsibility for the integrity of the data and the accuracy of the data analysis.

## Ethics Statement

This retrospective study was conducted in accordance with the Declaration of Helsinki and was approved by the Institutional Review Board of the First People′s Hospital of Yunnan Province (Approval Number KHLL2025‐KY155). The requirement for individual informed consent was waived due to the retrospective nature of the study, and all patient data were anonymized prior to analysis.

## Conflicts of Interest

The authors declare no conflicts of interest.

## Data Availability

The datasets used and/or analyzed during the current study are available from the corresponding authors on reasonable request.
